# Global trends in intestinal flora and ulcerative colitis research during the past 10 years: A bibliometric analysis

**DOI:** 10.3389/fmicb.2022.1003905

**Published:** 2022-09-07

**Authors:** Lu Zhang, Shuai Xiong, Fengchen Jin, Fan Zhou, Hongjun Zhou, Jinhong Guo, Chuanbiao Wen, Biao Huang

**Affiliations:** ^1^Chengdu University of Traditional Chinese Medicine, Chengdu, China; ^2^North Sichuan Medical College, Nanchong, China; ^3^Affiliated Hospital of Jiangxi University of Traditional Chinese Medicine, Nanchang, China

**Keywords:** intestinal flora, ulcerative colitis, trends, CiteSpace, VOSviewer

## Abstract

**Background and aim:**

Ulcerative colitis is a chronic inflammatory bowel disease, and intestinal flora plays an important role in ulcerative colitis. In this study, we conducted a bibliometric analysis of publications in the field of intestinal flora and ulcerative colitis research in the past 10 years to summarize the current status of the field and analyze the trends in the field.

**Methods:**

On July 15, 2022, we chose the Web of Science Core Collection database as the study’s data source. CiteSpace.5.8.R3 and VOSviewer 1.6.17 were used to examine publications of research on intestinal flora and ulcerative colitis that were published between 2012 and 2021. We looked through the papers for journals, organizations, nations and regions, authors, and key terms.

**Results:**

This analysis covered a total of 2,763 papers on studies into intestinal flora and ulcerative colitis. There were 13,913 authors, 93 nations, 3,069 organizations, and 759 journals in all of the articles. In the USA, 767 publications were the most. The university with the most publications was Harvard Medical School. The author with the most articles was Antonio Gasbarrini.

**Conclusion:**

This study summarizes the global research trends in intestinal flora and ulcerative colitis. Publications in this field have increased year by year in the last decade and the field of research on intestinal flora and ulcerative colitis has good prospects for growth.

## Introduction

Ulcerative colitis is a chronic inflammatory bowel disease, the pathogenesis of which is still unclear (Sonnenberg and Siegmund, 2016). The typical trait of ulcerative colitis is diffuse mucosal inflammation confined to the colonic region (Fell et al., 2016). Ulcerative colitis presents with bloody diarrhea, abdominal pain, fecal incontinence and fatigue (Segal et al., 2021). The incidence and prevalence of ulcerative colitis is highest in North America and Northern Europe. The incidence of ulcerative colitis is bimodal in character, with the first peak between the ages of 15–30 years and the second peak between the ages of 50–70 years (Burisch and Munkholm, 2015). Treatment of ulcerative colitis includes corticosteroids (Rhen and Cidlowski, 2005), aminosalicylates (Habens et al., 2005) and immunosuppressive agents (van Dieren et al., 2006). The etiology of ulcerative colitis involves interactions between the environment, the immune system, the gut microbiota, and genetic susceptibility to disease (Kobayashi et al., 2020). Imbalance of the intestinal flora can lead to intestinal inflammation. Recent studies have highlighted the role of intestinal flora in ulcerative colitis (Khan et al., 2019). Regulation of intestinal flora can treat ulcerative colitis (Damaskos and Kolios, 2008).

In bibliometrics, publications in a certain topic are quantitatively analyzed using statistical techniques (Ellegaard and Wallin, 2015). In 1969, American academics developed bibliometric analysis (Ma C. 2021). Researchers may easily understand the trends in their field of study with the aid of bibliometrics (Ma D. 2021). It evaluates a field’s state in terms of nations or regions, writers, institutions, etc. Numerous domains, such as cancer (Wang et al., 2021), pain (Luo et al., 2021), and infectious illnesses (Yang et al., 2020), have used bibliometrics. However, there has not been any bibliometric analysis done in the study on intestinal flora and ulcerative colitis. A bibliometric analysis of studies on intestinal flora and ulcerative colitis is necessary. We conducted a bibliometric analysis of publications on intestinal flora and ulcerative colitis from 2012 to 2021 with the intention of understanding the research trends in the area of intestinal flora and ulcerative colitis research during the last 10 years. We will summarize the current state of the field and analyze the trends in the field.

## Materials and methods

### Data collection and retrieval strategies

**Figure 1:**
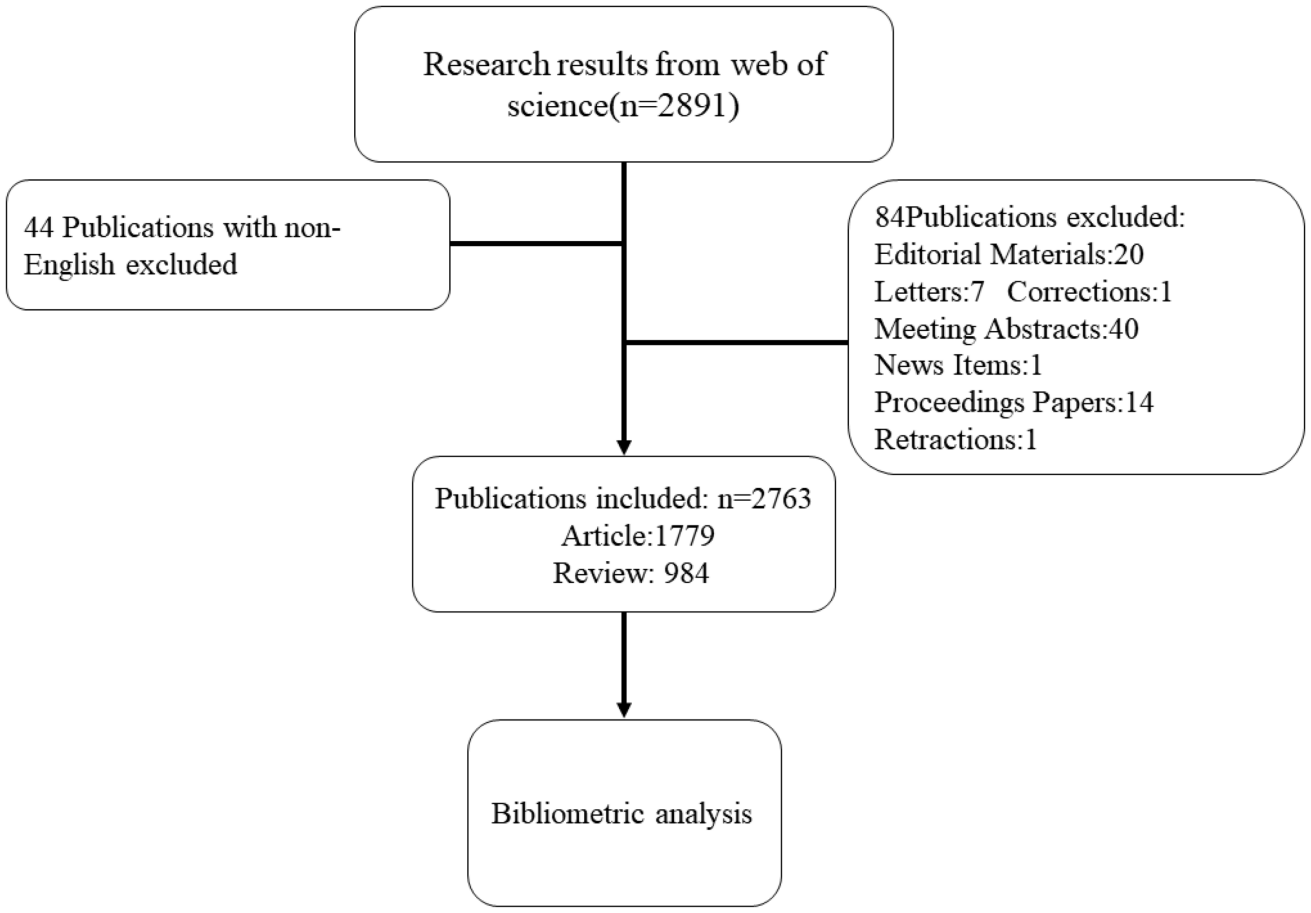
Flow chart of the study.

We obtained information from Clarivate Analytics’ Web of Science Core Collection (WoSCC) database. We were able to accurately analyze the papers since the WoSCC offers more information than other databases (Ma et al., 2022). The Social Sciences Citation Index (SSCI), Arts and Humanities Citation Index (A&HCI), Conference Proceedings Citation Index—Social Sciences and Humanities (CPCI-SSH), and Emerging Science Citation Index were among the versions of WoSCC that we searched (ESCI). Topic = (“gastrointestinal microbiome*” or “gut microbiome*” or “gut microflora” or “gut microbiota” or “gastrointestinal flora” or “gastrointestinal microbial communit* “or “gastrointestinal microflora” or “gastric microbiome*” or “intestinal microbiome*” or “intestinal flora” “gastrointestinal microbial communit* “OR “gastrointestinal flora” OR “gastrointestinal microbiota*”) AND Topic = (“ulcerative colitis” or “ulcerative colitis” or “ulcerous colitis “or “ulcerative colonitis” or “colitis ulcerosa” or “idiopathic proctocolitis” or “colitis gravis”). The article must be published between 2012-01-01 and 2021-12-31. The article can only be read in English. Only Article and Review articles could be found, and two researchers independently conducted the search. To lessen the bias brought on by automated database updates, the search was finished on July 15, 2022. Figure 1 depicts the literature screening procedure.

### Data analysis

To analyze the data from the literature, we utilized VOSviewer 1.6.17 and CiteSpace 5.8.R3. The literature’s authors, organizations, keywords, and journals were examined. CiteSpace’s specifications were configured, including the number of years in each slice (slice length = 1) and time slices from January 2012 to December 2021. The phrase “top 50 levels” is used as the threshold for the most commonly mentioned or cited in the relevant time slice, and all choices in the terminology source are verified. One node type is then chosen at a time based on particular criteria.

## Results

### Analysis of publication trends

**Figure 2:**
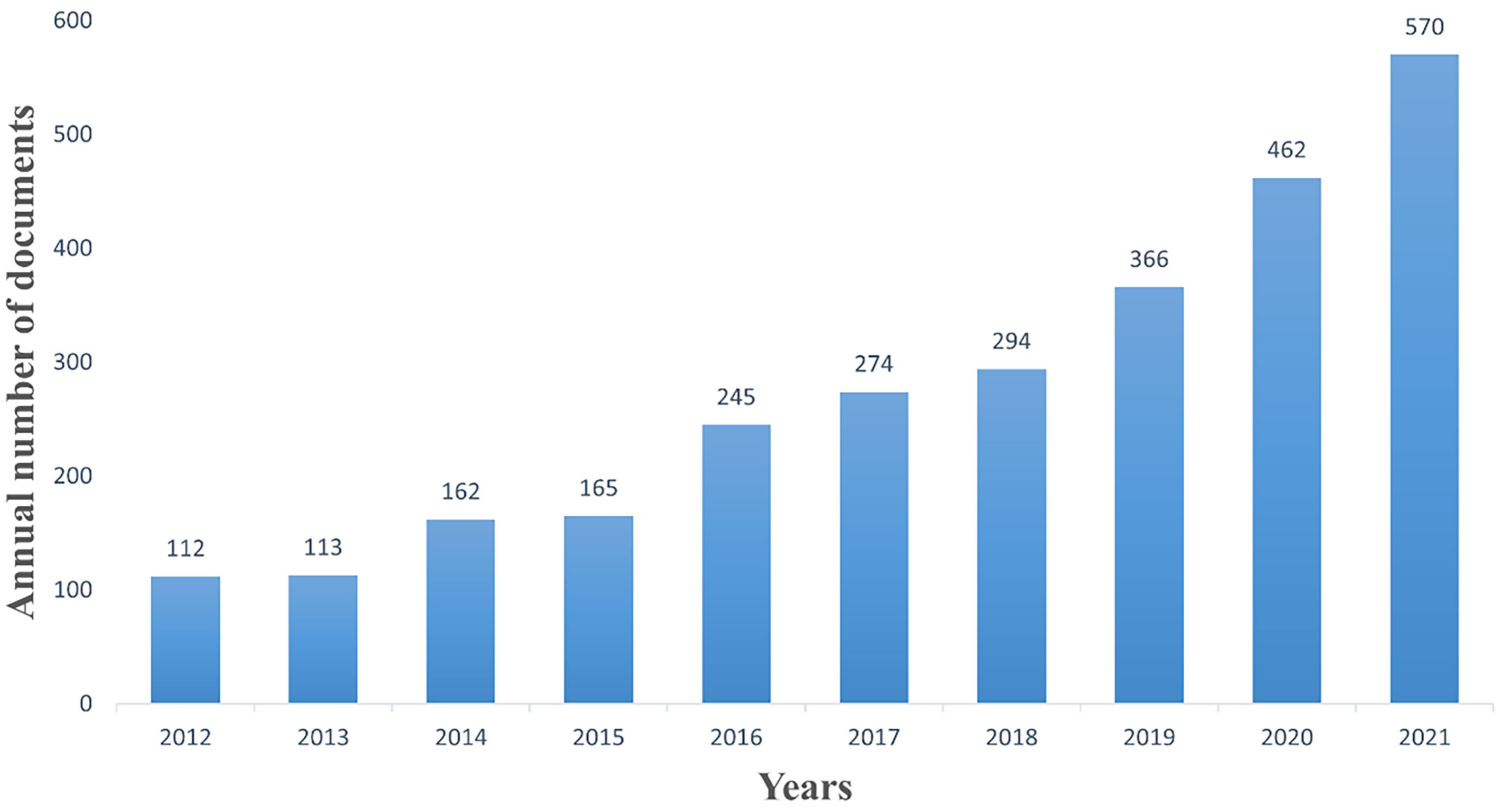
Trends in publications.

Finally, we incorporated 2,763 papers, comprising 1779 articles and 984 reviews, on research into intestinal flora and ulcerative colitis. Figure 2 depicts a general upward trend in the number of publications on research into intestinal flora and ulcerative colitis from the years 2012 to 2021. Between 2016 and 2018, there were 200 and 300 yearly publications. The number of yearly publications grew from 366 to 570 between 2019 and 2021.

### Analysis of the contribution of major countries

Between 2012 and 2021, 93 nations will participate in research on gut flora and ulcerative colitis. Table 1 lists the top 10 nations in the previous 10 years for research on intestinal flora and ulcerative colitis publications. The two biggest contributions were the United States and China. China came in second with 621 publications, trailing the United States with 767. Three nations made up the Asian region: China, Australia, and Japan. Italy, the United Kingdom, Germany, France, and Netherlands are among the five countries in Europe. The United States and Canada are located in the Americas. The strength of the cooperation may be shown by centrality. The United Kingdom, Canada, and Netherlands have the highest centrality of 0.15 out of the top 10 nations.

**Table 1:** The ten countries with the most publications.

Ranking	Countries	Centrality	Year	Publications
1	United States	0.06	2012	767
2	China	0.00	2012	621
3	Italy	0.12	2012	197
4	England	0.15	2012	184
5	Canada	0.15	2012	180
6	Germany	0.00	2012	148
7	Australia	0.00	2012	129
8	Japan	0.06	2012	119
9	France	0.03	2012	109
10	Netherlands	0.15	2012	87

### Analysis of major institutions

**Figure 3:**
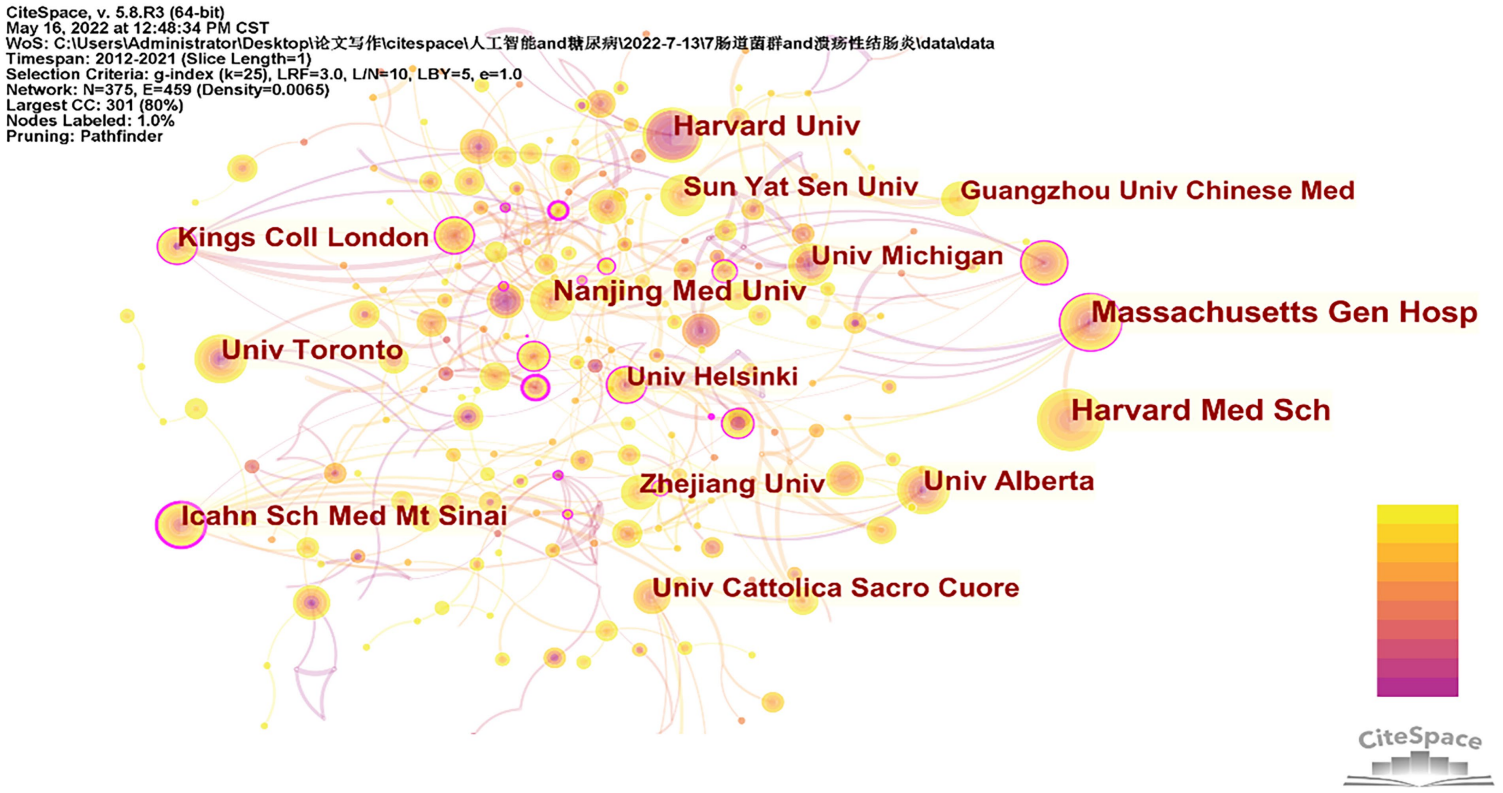
Institutional cooperation network.

A total of 3,069 institutions were involved in studies related to intestinal flora and ulcerative neo-colitis from 2012 to 2021. The 10 institutions with the highest number of publications are counted in Table 2. Those with > 30 publications were Harvard Medical School (*n* = 47), Massachusetts Hospital (*n* = 43), Harvard University (Walker et al., 2011), Nanjing Medical University (Kedia et al., 2021), University of Toronto (Cammarota et al., 2015), University of Alberta (*n* = 31) and Icahn School of Medicine at Mount Sinai (*n* = 31). Figure 3 shows the network of collaborative relationships among the major publishing institutions in this field. Larger centrality indicates stronger collaborative relationships at that institution. Icahn School of Medicine at Mount Sinai has the largest centrality of 0.24, followed by Massachusetts State Hospital at 0.16.

**Table 2:** Top ten institutions with the most publications.

Ranking	Institution	Centrality	Year	Publications
1	Harvard Medical School	0.00	2016	47
2	Massachusetts General Hospital	0.16	2012	43
3	Harvard University	0.04	2012	36
4	Nanjing Medical University	0.03	2016	35
5	University of Toronto	0.09	2012	32
6	University of Alberta	0.04	2012	31
7	Icahn School of Medicine at Mount Sinai	0.24	2014	31
8	Sun Yat-sen University	0.01	2015	28
9	Università Cattolica del Sacro Cuore	0.03	2014	26
10	Zhejiang University	0.01	2012	26

### Analysis of the main authors

**Figure 4:**
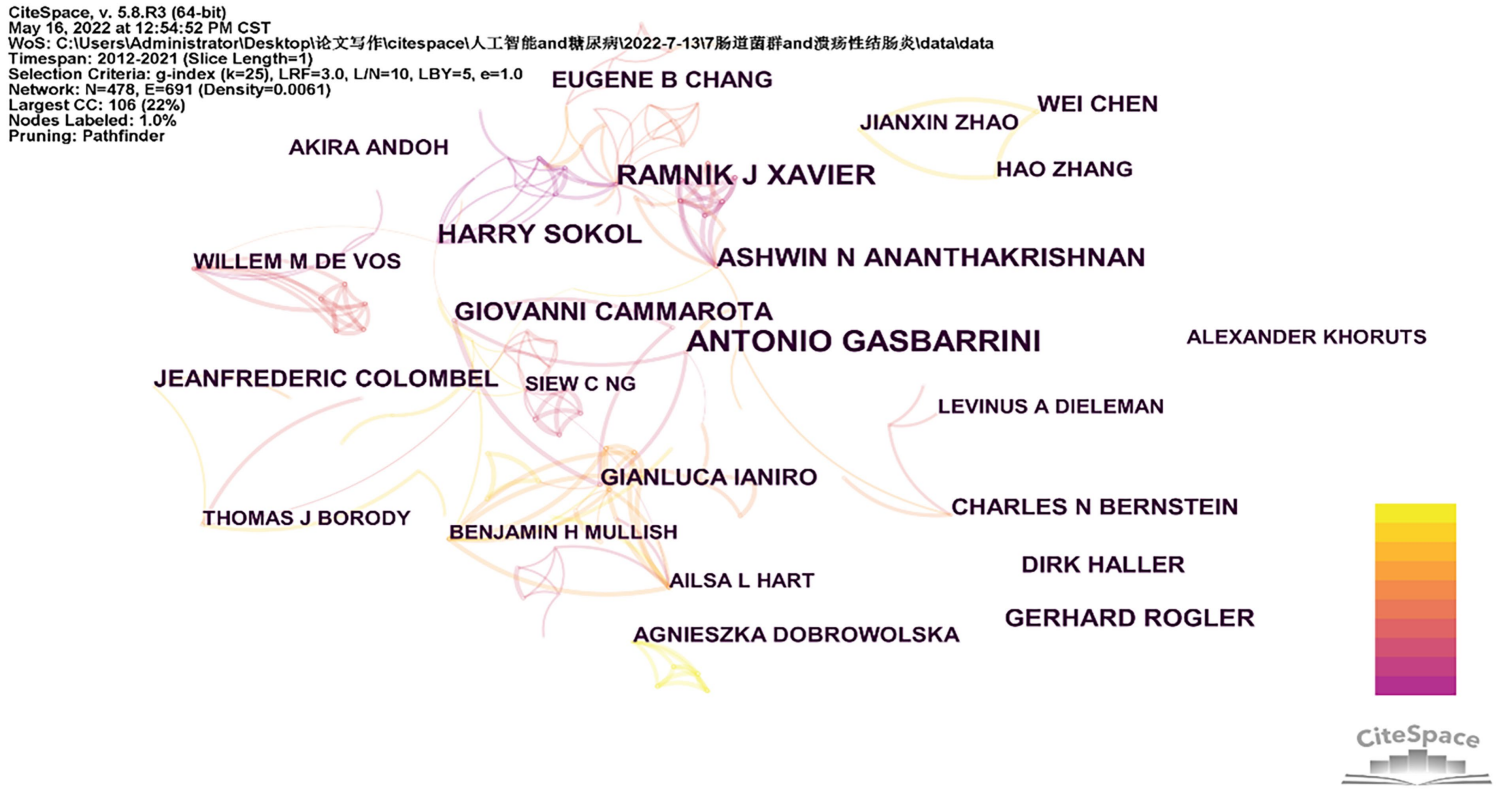
Author collaboration network.

Between 2012 and 2021, research on intestinal flora and ulcerative colitis had 13,913 authors in total. Table 3 includes a list of the top ten writers based on publications. Four of them, including Antonio Gasbarrini (Sokol et al., 2009), Ramnik J. Xavier (Marchesi et al., 2016), Ashwin N. Ananthakrishnan (Ma et al., 2022), and Harry Sokol (Ma et al., 2022), have published more than 15 publications. Figure 4 depicts the network of relationships that the leading authors in this subject have with one another. Collaboration exists between Jeanfrederic Colombel and Thomas J Borody. Benjamin H Mullish and Ailsa L Hart are working together. Hao Zhang, Wei Chen, and Jianxin Zhao work together on projects.

**Table 3:** The ten authors with the highest number of articles.

Ranking	Author	Centrality	Year	Publications
1	Antonio Gasbarrini	0.02	2014	29
2	Ramnik J Xavier	0.05	2012	21
3	Ashwin N Ananthakrishnan	0.03	2013	17
4	Harry Sokol	0.04	2012	17
5	Giovanni Cammarota	0.03	2014	15
6	Gerhard Rogler	0.00	2014	14
7	Jeanfrederic Colombel	0.00	2017	13
8	Eugene B Chang	0.00	2013	12
9	Wei Chen	0.00	2019	12
10	Gianluca Ianiro	0.00	2014	11

### Keyword analysis

**Figure 5:**
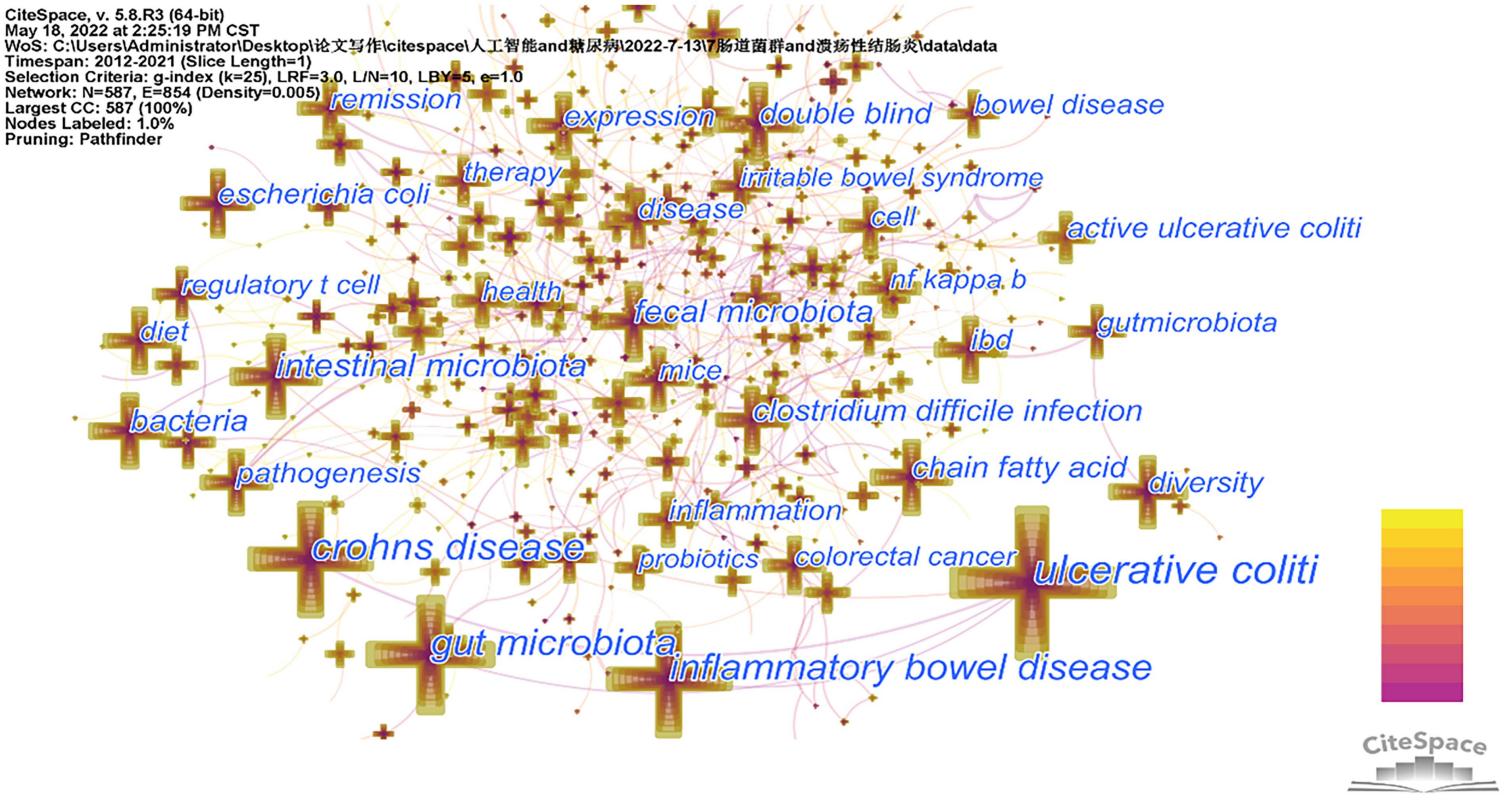
Keyword co-occurrence visualization.

**Figure 6:**
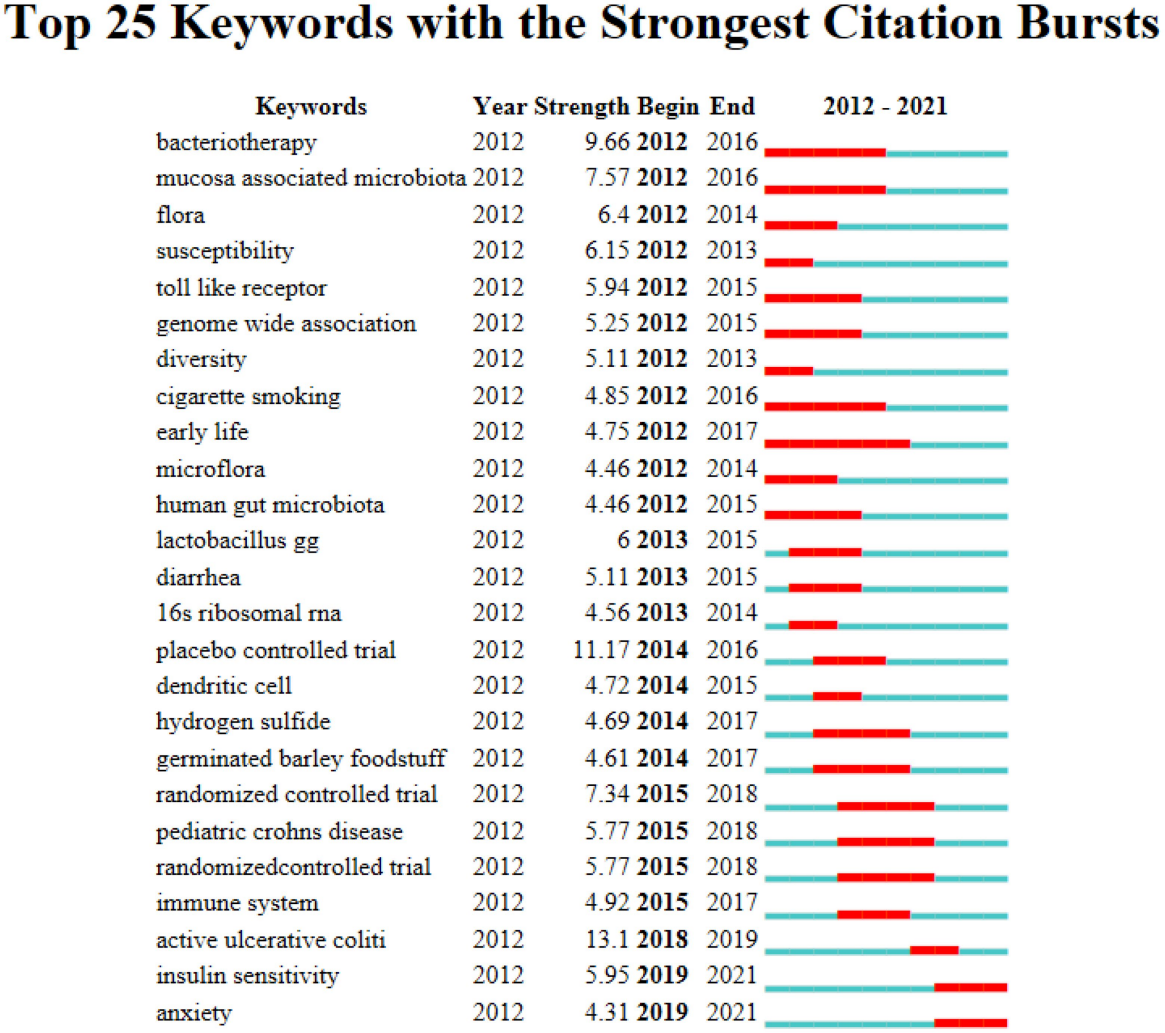
Keyword burst chart.

**Figure 7:**
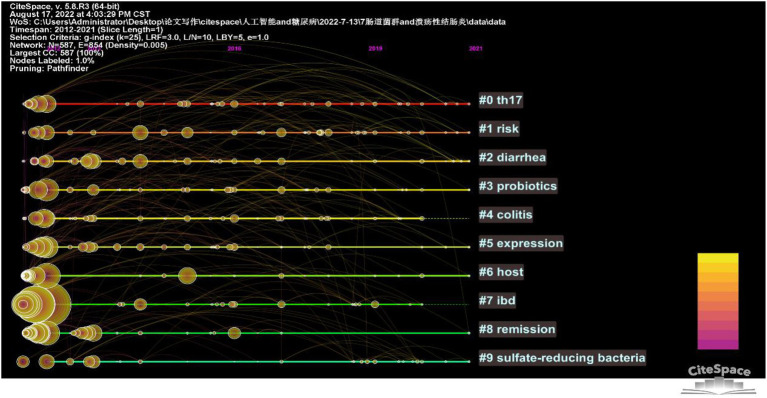
Keyword clustering timeline graph.

The 7,808 keywords were found in the 2,763 papers on intestinal flora and ulcerative colitis research during the last 10 years. The top 20 terms in this field by frequency are listed in Table 4. There are four of them that have frequencies > 300, including the terms “ulcerative colitis,” “crohn’s disease,” “inflammatory bowel illness,” and “gut microbiota.” Figure 5 shows the co-occurrence network of keywords with frequencies over 100 in studies related to gut microbiota and ulcerative colitis. The 25 terms in the field with the greatest epidemic intensity are listed in Figure 6. Active ulcerative colitis, insulin sensitivity, and anxiety are a few of the epidemic keywords that started to surface after 2018. Figure 7 shows the relationship between keyword clustering and time. The top ten keyword clusters in the field of intestinal flora and ulcerative colitis research are “#0 th17,” “#1 risk,” “#2 diarrhea,” “#3 probiotics,” “#4 colitis,” “#5 expression,” “#6 host,” “#7 ibd,” “#8 remission” and “#9 sulfate-reducing bacteria.”

**Table 4:** The 20 keywords with the highest frequency.

Ranking	Keywords	Centrality	Year	Count
1	ulcerative colitis	0.02	2012	1,471
2	crohn’s disease	0.00	2012	781
3	inflammatory bowel disease	0.00	2012	764
4	gut microbiota	0.00	2012	754
5	intestinal microbiota	0.03	2012	334
6	fecal microbiota	0.00	2012	265
7	bacteria	0.00	2012	218
8	chain fatty acid	0.04	2012	206
9	clostridium difficile infection	0.07	2012	188
10	double blind	0.01	2012	186
11	active ulcerative colitis	0.00	2015	179
12	ibd	0.03	2012	173
13	diversity	0.02	2012	164
14	expression	0.05	2012	160
15	*Escherichia coli*	0.00	2012	156
16	pathogenesis	0.03	2012	148
17	remission	0.05	2012	147
18	inflammation	0.05	2012	147
19	disease	0.21	2012	142
20	mice	0.06	2012	131

### Analysis of high yielding journals

**Figure 8:**
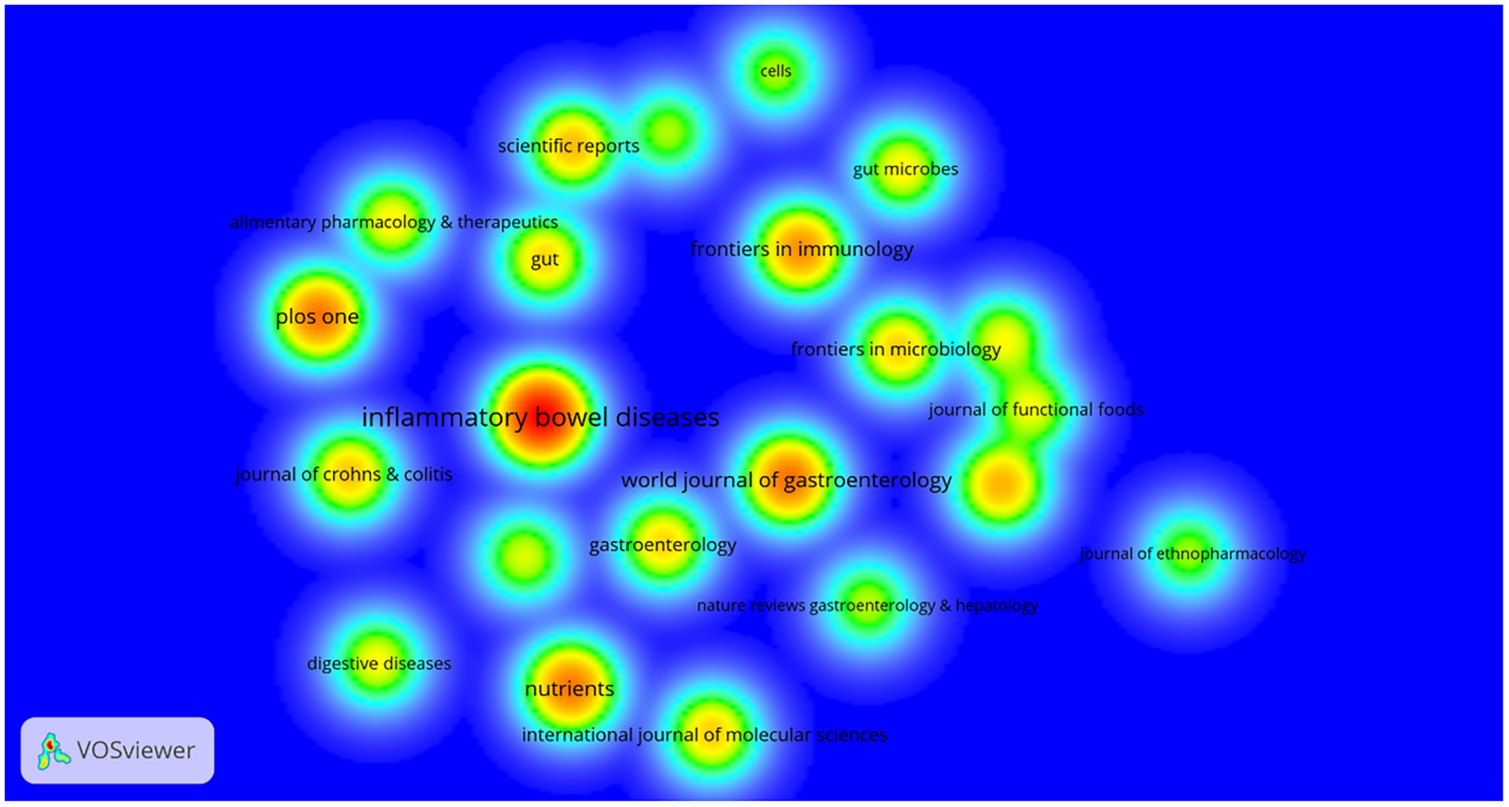
Journal density map.

Figure 8 lists the journals with less than 20 publications out of the total 2,763 papers on intestinal flora and ulcerative colitis research that were published in 759 journals worldwide between 2012 and 2021. Table 5 lists the ten journals that have published the most papers in this topic. Inflammatory Bowel Diseases (*n* = 123), World Journal of Gastroenterology (*n* = 69), Plos One (*n* = 68), Nutrients (*n* = 67), and Frontiers in Immunology (*n* = 58) were five journals with less than 50 articles. Inflammatory Bowel Diseases (*n* = 4,648), World Journal of Gastroenterology (*n* = 3,498), and Plos One (*n* = 3,453) were three journals with less than 3,000 citations each.

**Table 5:** Ten top journals.

Ranking	Journal	Citations	Publications
1	Inflammatory Bowel Diseases	4,648	123
2	World Journal of Gastroenterology	3,498	69
3	Plos one	3,453	68
4	Nutrients	2,029	67
5	Frontiers in Immunology	2,809	58
6	Food and Function	514	46
7	Scientific Reports	1,238	44
8	Journal of Crohn’s and Colitis	1,557	41
9	Frontiers in Microbiology	1,721	39
10	International Journal of Molecular Sciences	612	39

### Analysis of highly-cited papers

From 2012 to 2021, there were 2,763 articles on the topic of gut microbiota and ulcerative colitis. Of those, 23 studies had more than 500 citations. Table 6 displays the top ten articles in this field with the most citations. Four of them have citations in more than 1,000 publications. Include the article “The function of short-chain fatty acids in the interaction between food, gut microbiota, and host energy metabolism,” by den Besten et al. (2013), which has 2070 citations in the Journal of Lipid Research. Clemente et al. (2012) article “The influence of the gut microbiota on human health: an integrated picture” from the journal Cell was mentioned in 1961. The article “Dysfunction of the intestinal microbiota in inflammatory bowel disease and therapy” by Morgan et al. (2012) had 1,576 citations. With 1,132 citations, Marchesi et al. (2016) published “The gut microbiota and host health: a new clinical frontier” in the Gut Journal.

**Table 6:** Ten highly cited articles.

Rank	Title	Journals	First author	Year	Citations
1	The role of short-chain fatty acids in the interplay between diet, gut microbiota, and host energy metabolism	Journal of Lipid Research	Den Besten	2013	2,070
2	The impact of the gut microbiota on human health: an integrative view	Cell	Clemente	2012	1,961
3	Dysfunction of the intestinal microbiome in inflammatory bowel disease and treatment	Genome Biology	Morgan	2012	1,576
4	The gut microbiota and host health: a new clinical frontier	Gut	Marchesi	2016	1,132
5	The microbiome in inflammatory bowel disease: current status and the future ahead	Gastroenterology	Kostic	2014	971
6	The microbial metabolite butyrate regulates intestinal macrophage function *via* histone deacetylase inhibition	Proceedings of the National Academy of Sciences of the United States of America	Chang	2014	939
7	Short Chain Fatty Acids (SCFAs)-mediated gut epithelial and immune regulation and its relevance for inflammatory bowel diseases	Frontiers in Immunology	Parada Venegas	2019	833
8	Crohn’s disease	Lancet	Torres	2017	810
9	Epidemiology and risk factors for IBD	Nature Reviews Gastroenterology and Hepatology	Ananthakrishnan	2015	760
10	Multi-omics of the gut microbial ecosystem in inflammatory bowel diseases	Nature	Lloyd-price	2019	733

## Discussion

It is crucial to research gut flora and ulcerative colitis. The bibliometric analysis of works in this topic has never been done before. We considered a total of 2,763 papers from 2012 to 2021 that dealt with studies on intestinal flora and ulcerative colitis. We discovered that there are more research being conducted in this area each year. In 2019, there were more publications than 300. One of the reasons of ulcerative colitis, according to theory, is dysbiosis of the gut flora (Venegas et al., 2019). The rise in publications indicates that this field of study is currently one of the most popular research hot topics.

From 2011 to 2021, the United States and China made the most progress in the field of research on intestinal flora and ulcerative colitis. 767 publications total, with 621 coming from China, were the most. Researchers from the United States examined the reduced variety and richness of gut flora in children with ulcerative colitis (Michail et al., 2012). In children with ulcerative colitis, the gut microbiome’s composition and temporal alterations are linked to the disease process (Schirmer et al., 2018). Ursolic acid has been investigated by Chinese researchers to control the intestinal microbiota and inflammatory cell infiltration to avoid ulcerative colitis (Sheng et al., 2021). In ulcerative colitis patients, there was a reduction in beneficial bacteria and an increase in dangerous bacteria (He et al., 2021). In Xinjiang Uyghur, China, ulcerative colitis patients have lower levels of Clostridium, Bifidobacterium, Fusarium, and Proteus than healthy people (Yao et al., 2016). Harvard Medical School was the organization with the most amount of research papers on intestinal flora and ulcerative colitis published in this area47. The United States created the esteemed medical institution known as Harvard Medical School. DNA sequencing was used by researchers at Harvard Medical School to examine the gut flora in ulcerative colitis (Morgan et al., 2012). Changes in the gut flora can forecast how ulcerative colitis will develop (Ananthakrishnan et al., 2017). With 123 articles, Inflammatory Bowel Diseases is the journal with the most publications in this area. Prausnitzii in Colitis Microbiota” (Sokol et al., 2009) and “Phylogenetic Analysis of Dysbiosis in Ulcerative Colitis During Remission” (Rajilic-Stojanovic et al., 2013). ANTONIO GASBARRINI, who has published the most articles on intestinal flora and ulcerative colitis, has investigated the beneficial effects of antibiotics in regulating intestinal flora (Ianiro et al., 2016). In the treatment of ulcerative colitis, ciprofloxacin is crucial (Cammarota et al., 2015).

The high frequency keywords in this field are “chain fatty acid” and “clostridium difficile infection.” Intestinal flora and ulcerative colitis research both benefit from an understanding of chain fatty acids (Binder, 2010). According to research by laserna-Mendieta et al., a decline in chain fatty acids may be related to the onset of ulcerative colitis (Laserna-Mendieta et al., 2018). Anaerobic gut microbes fermenting generate chain fatty acids. Interestingly, ulcerative colitis has a diversified gut flora (Kedia et al., 2021). In acute ulcerative colitis, the gut flora is extremely unstable. In contrast to individuals with inflammatory bowel disease, the makeup of the intestinal flora fluctuates over time in normal persons. The microbial makeup of intestinal mucosa and feces differs significantly (Walker et al., 2011). Patients with ulcerative colitis are susceptible to clostridium difficile infection (Ananthakrishnan et al., 2013). Clostridium difficile infection is a gastrointestinal disease caused by Clostridium difficile, a Gram-positive, bacteriophage and toxin-producing anaerobic bacillus (Almeida et al., 2016). *Escherichia coli* may induce ulcerative colitis in immunosuppressed hosts or when the natural gastrointestinal barrier is impaired (Darfeuille-Michaud and Colombel, 2008). The growth of research has enhanced the study of gut flora and ulcerative colitis.

Our one bibliometric analysis of the field of intestinal flora and ulcerative colitis, like other bibliometric studies, has some limitations. Our data were derived from the Web of Science Core Collection database, and automatic updates of the database can affect differences in data volume. In general, the trends in the field will not change much.

## Conclusion

For this study, we used CiteSpace.5.8.R3 and VOSviewer1.6.17 to evaluate the previous 10 years’ worth of papers on intestinal flora and ulcerative colitis. 93 nations, 3,069 organizations, 13,913 authors, and 759 journals were represented in all articles. In the United States, there may be a maximum of 767 publications. With the most articles, Harvard Medical School tops the list of institutions. With the most articles, Antonio Gasbarrini is the author. The last 10 years have seen a rise in publications in this area, and the future of research on intestinal flora and ulcerative colitis is promising.

## Data availability statement

The original contributions presented in the study are included in the article/Supplementary material, further inquiries can be directed to the corresponding authors.

## Author contributions

SX and LZ wrote the manuscript and it was then revised by CW and BH. Additionally, FJ and HZ conducted a literature review and data analysis. All authors contributed to the article and approved the submitted version.

## Funding

This work was supported by the National Natural Science Foundation of China (82174236), Jiangxi Provincial Natural Science Foundation Youth Fund (20202BAL216065), Jiangxi Provincial Education Department Science Program (GJJ201259), and Jiangxi Provincial Science and Technology Department (20212BAG70037).

## Conflict of interest

The authors declare that the research was conducted in the absence of any commercial or financial relationships that could be construed as a potential conflict of interest.

## Publisher’s note

All claims expressed in this article are solely those of the authors and do not necessarily represent those of their affiliated organizations, or those of the publisher, the editors and the reviewers. Any product that may be evaluated in this article, or claim that may be made by its manufacturer, is not guaranteed or endorsed by the publisher.
